# From consciousness to observation: rethinking what consciousness science explains

**DOI:** 10.3389/fpsyg.2026.1762212

**Published:** 2026-08-26

**Authors:** Mario S. Staller, Swen Koerner

**Affiliations:** 1 University for Police and Public Administration, Cologne, Germany; 2 Department of Training Pedagogy and Martial Research, German Sport University Cologne, Cologne, Germany

**Keywords:** observation of observation, reflexivity, systems theory, theory of consciousness, observational form

## 1 From “uneasy stasis” to observational formats

Cleeremans et al.'s (2025) review article offers a comprehensive, up-to-date self-description of consciousness science: it organizes the field's central theoretical approaches, reconstructs key methodological and institutional developments, and discusses the ethical, clinical, and societal implications of possible “success” in understanding and manipulating consciousness. We take up this invitation to self-reflection by foregrounding a systems-theoretical perspective on scientific observation. In this tradition, observation is not the passive mirroring of a pre-given object; it is the operation of drawing a distinction and indicating one side (e.g., Luhmann, 1990). This is consequential for consciousness science because the field does not first encounter “consciousness” as an unformed object that is then merely measured. Rather, it can study consciousness only by stabilizing it as an object of inquiry through particular distinctions, operational definitions, and measurement decisions. Consciousness thus becomes empirically tractable in and through the field's forms of observation; at the same time, these forms are not arbitrary, but constrained by established methods, evidential practices, institutional routines, and the reproducibility of experimental discriminations.

From this observation-theoretical perspective, the “uneasy stasis” diagnosed by Cleeremans et al. (2025, p. 2)—and the associated risk “that researchers talk past each other rather than to each other” (p. 2)—can be specified more precisely. Our central claim is that this uneasy stasis can persist even under improved experimental rigor because competing programmes stabilize different evidence formats. By “evidence formats” we mean the bundle of commitments that makes empirical work possible in the first place: (i) the specification of the explanandum (what exactly is to be explained), (ii) the selection of accepted indicators (reports, behavior, neural signatures, and physiological measures), and (iii) the inferential rules that connect observed outcomes to claims about consciousness (what counts as supportive, disconfirming, or decisive evidence). Each evidence format also has an exclusion range: questions it cannot, in principle, decide under its assumptions and operationalisations. Methodological improvements typically increase precision within a format—through better controls, larger samples, or more reliable measurement—without aligning formats across programmes. Disagreement can therefore remain stable not because the field is careless, but because programmes generate increasingly precise results that are only limitedly comparable at the level of their formative commitments.

Making evidence formats explicit is therefore a methodological intervention. It clarifies what a result—given its operationalisation—actually supports, and what it cannot decide—given its exclusion range. In this sense, results can be “brought into dialogue” through explicit format specification that reveals where programmes are in fact answering different questions and, where possible, enable comparison conditions that align explananda, indicators, and inference rules. To operationalize this, we propose a simple template (explanandum, indicators, inference rule, exclusion range) and illustrate it with worked examples from standard paradigms in consciousness science. At the same time, we highlight a structural tension: progress depends on stabilizing formats that render consciousness testable, while these same formats generate systematic underdetermination at their boundaries.

## 2 Observation as distinction and indication: a systems-theoretical starting point

Cleeremans et al. (2025) situate contemporary consciousness science in a tradition that treats consciousness as a biological problem and locates its central task in understanding the biophysical basis of consciousness. This stance is a productive default within empirical sciences; our point is not to reject it based on our systems-theoretical perspective, but to make explicit the observational premises and consequences it brings with it in consciousness research. Systems theory describes science as a communicative system with its own evidential and observational (e.g., Luhmann, 1990, 2008; Stichweh, 1999). Empirical work, on this view, never begins with “raw” data, but with stabilized distinctions and operationalisations that define what can count as evidence in the first place.

The consequences of these premises therefore concern not only what becomes decidable, but also what remains invisible within a given format—its exclusion range—and thus the forms of disagreement and underdetermination that persist across programmes. Mechanistic programmes, for example, often presuppose that consciousness is an ontological property that is causally accessible, measurable, and modulable through intervention. This format makes many questions tractable, yet it can also render less visible alternative descriptions that emphasize the limits of external access, dependence on proxies, and the constructive role of operationalisations in producing an empirically workable target under methodological constraints. From an observation-theoretical standpoint, the point is not to settle this metaphysics, but to note: different forms of observation stabilize different objects-for-research.

This also makes the guiding question “Where is consciousness science going?” a structural one: which distinctions are privileged, which self-descriptions prevail, and which evidence formats become dominant in practice? Cleeremans et al. (2025) provide a rich answer at the level of content, but largely presuppose a particular observational form—namely, that consciousness is an ontological property that can be progressively characterized and tested. Our intervention brackets this ontological ambition and foregrounds the observational operation instead: what becomes visible about consciousness when we systematically track how the field's own distinctions and operationalisations shape its object and its criteria of progress?

On this view, familiar crisis points—for example, the “hard problem”, theoretical disputes, persistent methodological difficulties—appear not only as obstacles removable by better data, but also as expressions of format-based underdetermination. If programmes stabilize different explananda and different indicator–inference packages, disagreement can remain rational and persistent even under improved methods.

This dynamic is especially relevant in consciousness research because the field repeatedly confronts cases in which the attribution of consciousness is practically contested: no-report paradigms (Tsuchiya et al., 2015), disorders of consciousness (Luppi et al., 2021), and increasingly animal (Birch, 2022a,b), AI (Butlin et al., 2025) and general life/lyfe debates (Baluška and Reber, 2019; Bartlett and Wong, 2025). In such contexts, tests do not simply measure “a variable”; they instantiate evidence formats that stabilize attributions under uncertainty. This remains a methodological point: we do not claim that “social” considerations should determine scientific truth, but that scientific access to consciousness is mediated by observational formats—and that these formats constrain what can be said, compared, and decided.

## 3 Making observational formats explicit

One aim emphasized by Cleeremans et al. (2025) is to develop reliable tests for consciousness in humans, non-human animals, patients with disorders of consciousness, and potentially artificial systems. From the perspective developed here, such tests are core outputs of the field because they concretize evidence formats: they operationalize theoretical constructs, couple them to indicators (neural signatures, behavior, and reportability), and yield statistically evaluable results. Crucially, however, the meaning of a test does not lie in the data alone. Whether an outcome is treated as evidence for consciousness depends on prior format commitments—explanandum, accepted indicators, and inferential thresholds. Test results are therefore structured outputs of an evidence format, not unmediated readouts of an ontological property.

This becomes clear when standard paradigms are read in a format-explicit way. In contrastive fMRI designs comparing “conscious” vs. “unconscious” conditions, the usual interpretation as a neural correlates of consciousness (NCC) finding depends on what counts as “conscious” in the design (reportability, performance, subjective visibility, confidence, and post-decision access), which indicators are privileged, and which inference rule links the contrast to consciousness. The exclusion range then includes, among other things, whether the contrast isolates consciousness itself or rather reports metacognition, decision processes, attention, or task set. From our perspective, this does not diminish the value of such studies; it sharpens what they do—and do not—establish.

Similarly, clinical proxy measures in disorders of consciousness (e.g., perturbation complexity index, PCI) are useful precisely because they enable robust classifications under limited access (Casarotto et al., 2016); yet they may leave theory-level questions methodologically open. For example, in an IIT-oriented evidential frame (integrated information theory), the explanandum is consciousness as related to integration-like properties of a system's underlying cause–effect structure; PCI then functions as an IIT-inspired proxy, and high PCI values are treated as evidence that consciousness-relevant integration is present, even though PCI is neither a measure nor an approximation of Φ itself (Cleeremans et al., 2025; Tononi et al., 2016). In a GWT-oriented frame (global workspace theories), by contrast, the explanandum is conscious access or large-scale availability more broadly; here, PCI can be read as compatible with GWT without being theory-decisive.

In both cases, format specification increases comparability by making visible where programmes are genuinely addressing the same question and where they address different questions under different evidential conditions.

Against this background, recent methodological developments aimed at format alignment become more intelligible, including adversarial collaborations and consortium-based approaches (Consortium et al., 2025; Melloni et al., 2021). Meta-research suggests that “support” for theories can often be predicted from methodological choices, underscoring the need for explicit comparison conditions (Yaron et al., 2022). Specifically, this concerns choices such as whether studies target state or content consciousness, rely on report or no-report paradigms, privilege connectivity-based measures, or employ more subjective vs. objective indicators of consciousness. Accordingly, work on theory comparison stresses that meaningful tests require precise specification of the explanandum and comparison class, and alignment of predictions across theories (Chis-Ciure et al., 2024; Pin et al., 2021; Albantakis et al., 2023; Tononi et al., 2016). Current controversies around integrated information theory (IIT) show that disputes about testability, evidential standards, and decisive tests are, first of all, methodological disputes about evidence formats (Albantakis et al., 2023; Tononi et al., 2016). Recent exchanges make this especially clear: debates over whether PCI counts as a proxy, an approximation, or a genuinely theory-discriminating measure are not merely disagreements about one theory, but negotiations over what should count as admissible evidence in consciousness science (Gomez-Marin and Seth, 2025; IIT-Concerned et al., 2025). From our perspective, the demarcation dispute over IIT's “scientific” vs. “pseudoscientific” status is a sharpened expression of this more basic methodological conflict: a struggle over which observational and evidential frames the field is willing to stabilize as legitimate for theory-building and theory-testing (Gomez-Marin and Seth, 2025; IIT-Concerned et al., 2025; Tononi et al., 2025).

## 4 What would it mean to “get there”?

Where, then, is consciousness science going—and what would it mean to “get there”? From the perspective developed here, the field's future depends less on selecting a single “right” substantive theory than on increasing its reflexive capacity in handling observational and evidential formats. This includes making explicit programmes' guiding distinctions and exclusion ranges, understanding methodological innovations as stabilizing particular ways of observing, and acknowledging that advances in measurement and modeling do not automatically dissolve format pluralism. Success, on this view, would not be a format-free final verdict, but increasingly explicit, comparable, and well-scoped observational practices that state more clearly what can be decided—and what remains open under the given conditions.
